# Quality of breeding value predictions from longitudinal analyses, with application to residual feed intake in pigs

**DOI:** 10.1186/s12711-022-00722-w

**Published:** 2022-05-13

**Authors:** Ingrid David, Anne Ricard, Van-Hung Huynh-Tran, Jack C. M. Dekkers, Hélène Gilbert

**Affiliations:** 1 GenPhySE, INRAE, Université de Toulouse, INPT, 31326 Castanet Tolosan, France; 2 Université Paris Saclay, INRAE, AgroParisTech, GABI, 78352 Jouy-en-Josas, France; 3 Département Recherche et Innovation, Institut Français du Cheval et de l’Equitation, 61310 Exmes, France; 4 Department of Animal Science, Iowa State University, Ames, IA 50011 USA

## Abstract

**Background:**

An important goal in animal breeding is to improve longitudinal traits. The objective of this study was to explore for longitudinal residual feed intake (RFI) data, which estimated breeding value (EBV), or combination of EBV, to use in a breeding program. Linear combinations of EBV (summarized breeding values, SBV) or phenotypes (summarized phenotypes) derived from the eigenvectors of the genetic covariance matrix over time were considered, and the linear regression method (LR method) was used to facilitate the evaluation of their prediction accuracy.

**Results:**

Weekly feed intake, average daily gain, metabolic body weight, and backfat thickness measured on 2435 growing French Large White pigs over a 10-week period were analysed using a random regression model. In this population, the 544 dams of the phenotyped animals were genotyped. These dams did not have own phenotypes. The quality of the predictions of SBV and breeding values from summarized phenotypes of these females was evaluated. On average, predictions of SBV at the time of selection were unbiased, slightly over-dispersed and less accurate than those obtained with additional phenotypic information. The use of genomic information did not improve the quality of predictions. The use of summarized instead of longitudinal phenotypes resulted in predictions of breeding values of similar quality.

**Conclusions:**

For practical selection on longitudinal data, the results obtained with this specific design suggest that the use of summarized phenotypes could facilitate routine genetic evaluation of longitudinal traits.

**Supplementary Information:**

The online version contains supplementary material available at 10.1186/s12711-022-00722-w.

## Background

Selecting animals for a better feed efficiency is one of the key levers to improve farm profitability while reducing the environmental impact of livestock farming [1, 2]. Thanks to the development of electronic devices on farms, the recording of repeated phenotypes over time is facilitated in different livestock species [3–5]. This recording of longitudinal data is beneficial in a genetic context because it allows the extraction of useful information for selection on more complex criteria than the estimated breeding value (EBV) for the mean value of the trait over the studied period; thus, selection for an optimal shape of the curve [6–8] and/or for specific components of the curve, such as persistency of milk production [9] becomes possible. Longitudinal measurements are often correlated at both the genetic and environmental level, with, generally, a structured covariance pattern. To analyze such data with a limited number of parameters to be estimated, approaches that model the shape of the covariance functions, such as character process models (CP) [10], or that model the functions of the random effects, such as random regression (RR) [11, 12] or structured antedependence (SAD) [13] models, have been proposed. These models have proven to be efficient to model the covariance structure of the data and to accurately estimate breeding values for each time point in numerous studies [14–17]. However, these models are not widely used for routine genetic evaluation in livestock except for milk production traits in dairy cattle [6], for several reasons. First, combining time point EBV in a selection index remains an issue, and second, fitting these models is computationally demanding, especially when accounting for genomic information. To overcome these challenges, the use of eigenvectors of the genetic covariance matrix to linearly combine the time point breeding values into several summarized breeding values (SBV) has been proposed [18]. These eigenvectors have been shown to make sense biologically for several longitudinal traits [18–21] and allow predictions for independent SBV to be obtained for selection. In addition, the use of SBV results in fewer equations in the mixed model and better convergence properties [22].

Thanks to new genotyping technologies that render the genotyping of numerous single nucleotide polymorphisms (SNPs) cost-effective, genomic prediction (GP) has been implemented in multiple livestock species [23–28]. For example, genomic EBV (GEBV) can be obtained by single-step genomic best linear unbiased prediction (ssGBLUP), which combines in a single-step pedigree data, phenotypes, and genotypes for genetic evaluation [29]. The efficiency of genomic selection on these GEBV depends on their bias and accuracy [30]. Traditionally, the accuracy of GEBV is obtained by cross-validation approaches, based on the computation of correlations between adjusted phenotypes and GEBV of hypothetical selection candidates. This is, however, not straightforward for predictions of SBV. As an alternative, Legarra and Reverter [31] proposed the linear regression method (LR method), which provides a series of statistics to quantify the quality of predictions that can be used in complex scenarios where adjusted phenotypes or coefficients of determination are not straightforward to obtain, such as for SBV.

Thus, our goal was to propose and test operational solutions that could facilitate the implementation of selection for longitudinal traits, using the example of feed efficiency. Using the LR method, the objectives of the present study were to evaluate the quality of the predictions of SBV for longitudinal RFI in pigs, as well as the benefit of adding genomic information. In addition, for practical selection on longitudinal profiles, we evaluated the effect of using summarized phenotypes instead of longitudinal phenotypes on the quality of predictions of SBV.

## Methods

### Material

Phenotypes on feed intake (FI) and production (metabolic body weight (MBW), average daily gain (ADG), and backfat thickness (BFT)) records measured weekly on 2397 growing French Large White pigs over a 10-week period from ~ 13 to ~ 22 weeks of age were used in this study (descriptive statistics are in Additional file 1 Table S1). These animals were from seven generations of a divergent selection experiment for RFI applied at the end of each test period (110 kg) [1]. Animal management and phenotype measurements are described in David et al. [32] and Huynh-Tran et al. [19]. The numbers of animals and records per low (LRFI), high RFI (HRFI) lines and generation are in Additional file 1: Table S2. All sires and dams (660 pigs) of the phenotyped animals of generations G1 to G7 were genotyped using the Illumina SNP60 Beadchip V2 (64,232 SNPs) or the Illumina Porcine HD Array GGP (68,516 SNPs). None of the parents had own phenotypes. After quality control, genotyped or imputed genotypes for 64,487 SNPs on 624 pigs were available for further analyses (see Additional file 1: Table S2). The inverse of the $\mathbf{H}$ matrix that combines genomic and pedigree information was obtained using the method proposed by Legarra et al. [29] as $${\mathbf{H}}^{-1}={\mathbf{A}}^{-1}+\left[\begin{array}{cc}0& 0\\ 0& {{\varvec{\Omega}}}_{\upomega }^{-1}-{\mathbf{A}}_{22}^{-1}\end{array}\right]$$, where ${\mathbf{A}}_{22}$ is the pedigree-based numerator relationship matrix for the genotyped animals and ${{\varvec{\Omega}}}_{{\varvec{\upomega}}}=\left(1-0.05\right){{\varvec{\Omega}}}^{*}+0.05{\mathbf{A}}_{22}$. Matrix ${{\varvec{\Omega}}}^{*}$ was obtained by scaling the genomic relationship matrix ${\varvec{\Omega}}$ [33], to make the means of the diagonal and off-diagonal elements of ${{\varvec{\Omega}}}^{*}$ and ${\mathbf{A}}_{22}$ matrices equal to each other. The $\mathbf{A}$ matrix was obtained for all animals in the pedigree plus ancestors (grandparents), and comprised 3095 individuals over 10 generations. The $\mathbf{H}$ matrix was obtained and formatted using the PreGSf90 software [34] and modified with R to be supplied to ASReml as a user defined relationship matrix.

### Methods

Longitudinal production and FI records of all phenotyped animals (whole data) were used to compute longitudinal RFI by a phenotypic regression of FI on production and maintenance traits, using the following RR model of degree 2 for the genetic and permanent environmental effects:

$${FI}_{ij}=\,{\mathbf{x}}_{ij}{\varvec{\upbeta}}+{\beta}_{1}{MBW}_{ij}+{\beta}_{2}{ADG}_{ij}+{\beta }_{3}{BFT}_{ij}+\sum_{q=0}^{2}{\mathrm{a}}_{iq}{\varphi }_{q}\left(j\right)+\sum_{q=0}^{2}{\mathrm{b}}_{iq}{\varphi }_{q}\left(j\right)+{e}_{ij},$$

where ${FI}_{ij}$, ${MBW}_{ij}$, ${ADG}_{ij}$, and ${BFT}_{ij}$ are the FI, MBW, ADG and BFT of animal $i$ in week $j$, ${\beta }_{1}$, ${\beta }_{2}$, and ${\beta }_{3}$ are the phenotypic regression coefficients linking production and maintenance traits to FI, and ${\varphi }_{q}\left(j\right)$ is the ${\left(q+1\right)}^{th}$ Legendre polynomial at time $j$. Vectors $\mathbf{a}=\left({\mathbf{a}}_{\boldsymbol{1}},{\mathbf{a}}_{\boldsymbol{2}},\dots {\mathbf{a}}_{\boldsymbol{3095}}\right),\boldsymbol{ }{\mathbf{a}}_{{\varvec{i}}}=({\mathrm{a}}_{i0},{\mathrm{a}}_{i1},{\mathrm{a}}_{i2})$, and $\mathbf{b}=\left({\mathbf{b}}_{\boldsymbol{1}},{\mathbf{b}}_{\boldsymbol{2}},\dots {\mathbf{b}}_{\boldsymbol{2397}}\right),\boldsymbol{ }{\mathbf{b}}_{{\varvec{i}}}=({\mathrm{b}}_{i0},{\mathrm{b}}_{i1},{\mathrm{b}}_{i2})$ are the vectors of random coefficients for the genetic and permanent environmental effects, respectively, with $\mathbf{a}\sim N\left(\boldsymbol{0},\mathbf{A}\otimes {\mathbf{K}}_{{\varvec{a}}}\right)$ and $\mathbf{b}\sim N\left(\boldsymbol{0},\mathbf{I}\otimes {\mathbf{K}}_{{\varvec{b}}}\right)$, ${\mathbf{K}}_{{\varvec{a}}}$ and ${\mathbf{K}}_{{\varvec{b}}}$ being the 3 × 3 covariance matrices between genetic and permanent random coefficient, respectively and $\mathbf{e}$ is the residual vector, with heterogeneous variances over weeks ($\mathbf{e}\sim N(\boldsymbol{0},\mathbf{I}\otimes \mathbf{D})$, where $\mathbf{D}$ is a 10 × 10 diagonal matrix. Thus, the distribution of the additive genetic (${u}_{ij}=\sum_{q=0}^{2}{\mathrm{a}}_{iq}{\varphi }_{q}\left(j\right))$ and permanent environmental effects (${p}_{ij}=\sum_{q=0}^{2}{\mathrm{b}}_{iq}{\varphi }_{q}\left(j\right))$ are $\mathbf{u}\sim N\left(\boldsymbol{0},\mathbf{A}\otimes \mathbf{G}\right)$ and $\mathbf{p}\sim N\left(\boldsymbol{0},\mathbf{I}\otimes \mathbf{P}\right)$, respectively, with $\mathbf{G}={\varvec{\uppsi}}{\mathbf{K}}_{{\varvec{a}}}{\varvec{\uppsi}}\mathbf{^{\prime}}$, where ${\varvec{\uppsi}}$ is the (10 × 3) matrix of the Legendre polynomials for all time points and $\mathbf{P}={\varvec{\uppsi}}{\mathbf{K}}_{{\varvec{b}}}{\varvec{\uppsi}}\mathbf{^{\prime}}$. For selection purposes, the $10$ breeding values per animal (one for each time point) are summarized into a reduced number of values (SBV) that are interpretable and of interest for selection (i.e. that give information about the shape of the BV trajectory). These SBV were obtained by an Eigen decomposition of the genetic covariance matrix $\mathbf{G}$. The ${l}^{\mathrm{th}}$ SBV for animal $i$ is then ${\mathrm{SBV}}_{l,i}={\mathbf{L}}_{\mathrm{G}l}{\mathbf{u}}_{i}$, where ${\mathbf{L}}_{\mathrm{G}l}$ is the ${l}^{\mathrm{th}}$ eigenvector from the decomposition of the $\mathbf{G}$ matrix and ${\mathbf{u}}_{i}$ the vector of breeding values for animal $i$ (${\mathbf{u}}_{i}=({\mathrm{u}}_{i1},{\mathrm{u}}_{i2},\dots ,{\mathrm{u}}_{i10})$). Fixed effects included in the model (${\mathbf{x}}_{ij}{\varvec{\upbeta}}$) were the combination week by generation (10 × 8 levels), the combination batch by sex (66 × 3 levels), birth herd (2 levels), age at start of the test (covariate), and pen (16 levels). Variance components for this full dataset were estimated using the REML approach in ASReml 4.0 [35] and were considered as known in all subsequent analyses. Estimates of heritability of each ${SBV}_{l}$, obtained by the Eigen decomposition of the genetic covariance matrix $\mathbf{G}$, were computed as ${h}_{{SBV}_{l}}^{2}=\frac{{\mathbf{L}}_{\widehat{\mathbf{G}},{\varvec{l}}}^{\mathbf{^{\prime}}}\widehat{\mathbf{G}}{\mathbf{L}}_{\widehat{\mathbf{G}},{\varvec{l}}}}{{\mathbf{L}}_{\widehat{\mathbf{G}},{\varvec{l}}}^{\mathbf{^{\prime}}}\left(\widehat{\mathbf{G}}+\widehat{\mathbf{P}}+\widehat{\mathbf{D}}\right){\mathbf{L}}_{\widehat{\mathbf{G}},{\varvec{l}}}}$.

**Fig. 1:**
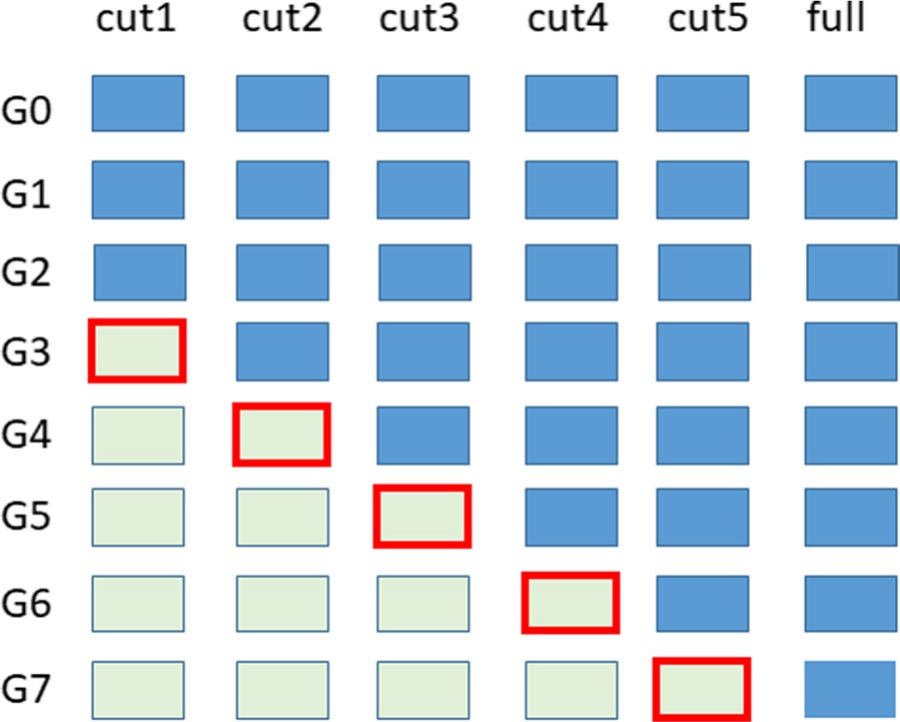
Scenarios retained to test the effect of additional phenotypic or genomic information on predictions, depending on the number of phenotyped generations. Blue box: animals with phenotypes, green box: animals without phenotypes, red indicates the progeny of the focal individuals genotyped (sires and dams of the previous generation that do not have own phenotype). For instance in cut1, focal animals are the genotyped sire and dams of G3 animals

The LR method applied to the SBV of a group of focal individuals (i.e. individuals of interest) consists in comparing the estimated SBV (ESBV) of these focal individuals based on less information to their ESBV based on more information, considering that more information provides a reasonable reference. ESBV of the focal individuals based on less information (partial) are referred to as ${ESBV}_{p}$ and their ESBV based on more information (whole) as ${ESBV}_{w}$. For the sake of simplicity, the subscript of SBV is not added, thus ESBV refer to the first, second or third ESBV, indifferently. Three of the five statistics based on the comparison of these two sets of ESBV, as proposed by Legarra and Reverter [31], were used here, each referring to properties of ${ESBV}_{p}$ for the focal individuals. The estimate of the standardized bias of ${ESBV}_{p}$ was computed as (for the $l$^th^ SBV) $\frac{\overline{{ESBV }_{p}}-\overline{E{SBV }_{w}}}{{\mathbf{L}}_{\widehat{\mathbf{G}},{\varvec{l}}}^{\mathbf{^{\prime}}}\widehat{\mathbf{G}}{\mathbf{L}}_{\widehat{\mathbf{G}},{\varvec{l}}}}$, where $\overline{{ESBV }_{p}}$ and $\overline{{ESBV }_{w}}$ are the mean of ${ESBV}_{p}$ and $E{SBV}_{w}$, respectively. Its expected value is 0 if the evaluation with the partial dataset is unbiased. Dispersion of ${ESBV}_{p}$ was evaluated by the regression of ${ESBV}_{w}$ on ${ESBV}_{p}$: ${b}_{w,p}=\frac{cov\left({ESBV}_{p},{ESBV}_{w}\right)}{var\left({ESBV}_{p}\right)}$. Its expected value is 1, if there is no over-/under-dispersion. Finally, the ratio of the prediction accuracy (i.e. the correlation between true and estimated breeding values) of ${ESBV}_{p}$ to the prediction accuracy of ${ESBV}_{w}$ was evaluated by the correlation between these ESBV: ${\rho }_{w,p}=\frac{cov\left({ESBV}_{p},{ESBV}_{w}\right)}{\sqrt{var\left({ESBV}_{p}\right)var\left(E{SBV}_{w}\right)}}$. The lower this correlation is, the higher is the relative gain in accuracy due to additional information. We evaluated the quality of SBV predictions for genotyped dams, which did not have own phenotypes (but had phenotyped half-sibs and full-sibs, on average 24 and 4 per dam, respectively), as focal individuals by comparing their SBV estimated with less and more information. The additional information considered were the phenotypes of their descendants or their genomic information. To do so, five cut-offs were considered, corresponding to the number of generations with phenotypes in the dataset (from 3 generations (G0, G1, G2, cut-1) to 7 generations (cut-5)), as described in Fig. 1. Based on these cut-offs, five groups of focal individuals were defined (group_FI$i$, $i$ = 1,…,5), which corresponded to the dams of the first generation of animals without phenotypes. For instance, the first group of focal individuals (group_FI1) consisted of the dams of the first generation without phenotypes in cut-1, i.e. the dams of the phenotyped animals in G3. For each group of focal individuals, the three LR statistics were computed to compare predictions obtained with less information to those obtained with more information. Thus, the following comparisons made were:

(1) SBV predicted for the group of focal individuals (group_FI$i$) obtained with phenotypes in cut_$i$ compared to SBV predicted for the group of focal individuals obtained with phenotypes in cut_($i$+1), cut_($i$+1) corresponding to the full dataset if ($i$ = 5) using (a) pedigree information only (i.e. $\mathbf{A}$ matrix) or (b) genomic information (i.e. the $\mathbf{A}$ matrix is then replaced by the $\mathbf{H}$ matrix for the distribution of genetic effects in the RR model);

(2) SBV predicted for the group of focal individuals (group_FI$i$) obtained with phenotypes in cut_$i$ compared to SBV predicted for the group of focal individuals obtained with phenotypes of the full dataset using (a) pedigree information only (i.e. $\mathbf{A}$ matrix)) or (b) genomic information (i.e. $\mathbf{H}$ matrix);

(3) SBV predicted for the group of focal individuals using pedigree information (i.e. $\mathbf{A}$ matrix) compared to SBV obtained with phenotypes of the same dataset using genomic information (i.e. $\mathbf{H}$ matrix). SBV were obtained using phenotypes in (a) cut_$i$ or (b) phenotypes of the full dataset.

The first two comparisons allowed us to evaluate the quality of the model to predict SBV based on phenotypic information on ascendants and collateral relatives of the focal individuals. For these two comparisons, two “whole” datasets were considered as the gold standard (cut-($i$+1) or full data) to account for random errors in the estimates of the LR statistics [36]. The third comparison allowed us to evaluate the benefit of genomic information for predictions.

The comparisons were performed separately for each line and for the three first SBV. These comparisons led to 108 estimates of the three LR statistics when evaluating the quality of the model to predict SBV based on phenotypic information on ascendants and collateral relatives of the focal individuals (2 lines, 3 SBV, $\mathbf{A}$ or $\mathbf{H}$ matrix, 5 cut-offs), and 30 estimates of the three LR statistics when evaluating the quality of the model to predict SBV based on phenotypic and pedigree information (gold standard being GSBV, 2 lines, 3 SBV, 5 cut-offs). Tests of the impact of the different factors (line, SBV, type of genetic information) on the LR statistics were evaluated using linear models including these three factors, and likelihood ratio tests. The two-by-two interactions between all factors were tested and kept in the model only when they were significant for one LR statistic.

Mirroring the SBV, fixed effects and variance component estimates obtained for the full dataset and the pedigree relationship matrix were used to compute the following three summarized phenotypes $\left({\mathbf{y}}_{{{\varvec{S}}{\varvec{B}}{\varvec{V}}}_{{\varvec{l}}}},\boldsymbol{ }l=\mathrm{1,2},3\right)$: ${\mathbf{y}}_{{{\varvec{S}}{\varvec{B}}{\varvec{V}}}_{{\varvec{l}}}}={\mathbf{L}}_{\widehat{\mathbf{G}},\mathbf{l}}^{\mathbf{^{\prime}}}\left(\mathbf{F}\mathbf{I}-\mathbf{X}\widehat{{\varvec{\upbeta}}}-\widehat{{\beta }_{1}}\mathbf{M}\mathbf{B}\mathbf{W}-\widehat{{\beta }_{2}}\mathbf{A}\mathbf{D}\mathbf{G}-\widehat{{\beta }_{3}}\mathbf{B}\mathbf{F}\mathbf{T}\right)$. In order to obtain summarized phenotypes for all animals for which one of the longitudinal phenotype (FI, MBW, ADG or BFT) was missing (4.2% of the data corresponding to 20% of the animals with at least one missing weekly phenotype), missing values for $\mathbf{F}\mathbf{I}-\mathbf{X}\widehat{{\varvec{\upbeta}}}-\widehat{{\beta }_{1}}\mathbf{M}\mathbf{B}\mathbf{W}-\widehat{{\beta }_{2}}\mathbf{A}\mathbf{D}\mathbf{G}-\widehat{{\beta }_{3}}\mathbf{B}\mathbf{F}\mathbf{T}$ were replaced by the average value per week of the full population before multiplying by ${\mathbf{L}}_{\widehat{\mathbf{G}},{\varvec{l}}}^{\mathbf{^{\prime}}}$. Breeding values for the summarized phenotypes were obtained using the following model: ${\mathrm{y}}_{{SBV}_{l},i}={\mu }_{l}+{u}_{{\mathrm{y}SBV}_{l},i}+{e}_{{SBV}_{l},i}$, with variance components derived from estimates obtained from the model for longitudinal RFI: ${{\varvec{u}}}_{{\mathbf{y}{\varvec{S}}{\varvec{B}}{\varvec{V}}}_{{\varvec{l}}}}\sim N\left(\boldsymbol{0},\mathbf{A}{\mathbf{L}}_{\widehat{\mathbf{G}},{\varvec{l}}}^{\mathbf{^{\prime}}}\widehat{\mathbf{G}}{\mathbf{L}}_{\widehat{\mathbf{G}},{\varvec{l}}}\right)$ and ${{\varvec{e}}}_{{{\varvec{S}}{\varvec{B}}{\varvec{V}}}_{{\varvec{l}}}}\sim N\left(\boldsymbol{0},\mathbf{I}{\mathbf{L}}_{\widehat{\mathbf{G}},{\varvec{l}}}^{\mathbf{^{\prime}}}\left(\widehat{\mathbf{P}}+\widehat{\mathbf{D}}\right){\mathbf{L}}_{\widehat{\mathbf{G}},{\varvec{l}}}\right)$. To further evaluate the applicability of longitudinal models for routine genetic evaluation, we then compared the EBV of ${\mathbf{y}}_{{{\varvec{S}}{\varvec{B}}{\varvec{V}}}_{{\varvec{l}}}}\boldsymbol{ }\left(\widehat{{\mathbf{u}}_{{\mathbf{y}{\varvec{S}}{\varvec{B}}{\varvec{V}}}_{{\varvec{l}}}}}\right)$ to those obtained for ${SBV}_{l}$, assuming that the eigenvectors do not have to be re-computed for each new evaluation.

## Results

The genetic parameters were estimated based on the full dataset and the pedigree relationship matrix. The three first eigenvalues from the eigen decomposition of $\widehat{\mathbf{G}}$ represented 59, 26 and 15% of the total genetic variance, respectively. Heritability estimates of the corresponding first three SBV were 0.36 ± 0.05, 0.20 ± 0.04, and 0.16 ± 0.05, respectively. The LR statistics and p-values of the different factors evaluating the quality of (G)ESBV obtained at the time of selection are summarized in Table 1. On average, ESBV were unbiased, slightly overdispersed and less accurate than ESBV predicted with more phenotypic information ($\overline{{\Delta \mu }_{wp}}=$ 0.00, 95% Confidence Interval: [− 0.01,0.01], $\overline{{b }_{w,p}}=$ 0.84 [0.79,0.89], and $\overline{{\rho }_{w,p}}=$ 0.61 [0.58, 0.64]). Overdispersion was significantly larger for the LRFI line than for the HRFI line (${b}_{w,p}$= 0.75 [0.68,0.82] versus 0.93 [0.86,1.00]) and for SBV3 compared to SBV2 (${b}_{w,p}$= 0.76 [0.69,0.85] versus 0.93 [0.85,1.00]) (see Additional file 2: Fig. S1). The ratio of accuracy between SBV obtained with more or less phenotypic information was significantly lower for SBV3 than for SBV2 (${\rho }_{w,p}$= 0.56 [0.51,0.61] versus 0.67 [0.61,0.72]). Considering the SBV obtained using pedigree and genomic information as the gold standard, on average, SBV obtained with pedigree information only were biased downwards ($\overline{{\Delta \mu }_{wp}}=$− 0.08 [− 0.09,− 0.07]). The bias differed between SBV: underestimated for SBV_1_ and SBV_2_ and overestimated for SBV_3_ (see Additional file 2: Fig. S2). Finally, the SBV predicted using longitudinal phenotypes and EBV predicted using summarized phenotypes ($\widehat{{\mathbf{u}}_{{\varvec{y}}{{\varvec{S}}{\varvec{B}}{\varvec{V}}}_{{\varvec{p}}}}}$) for genotyped animals in the full dataset are summarized in Table 2: the EBV obtained from summarized phenotypes were unbiased, neither over- nor under-dispersed, and were as accurate as the SBV predicted using longitudinal phenotypes, when computed with the pedigree information only ($\mathbf{A}$ matrix) or combining pedigree and genotypes ($\mathbf{H}$ matrix).

**Table 1:** Average LR statistics and p-values of the tested factors for the prediction of SBV

Partial data	Whole data			${\boldsymbol{\Delta }{\varvec{\mu}}}_{{\varvec{w}}{\varvec{p}}}$	${{\varvec{b}}}_{{\varvec{w}},{\varvec{p}}}$	${{\varvec{\rho}}}_{{\varvec{w}},{\varvec{p}}}$
No phenotypic information from candidates' descendants	With phenotypic information from candidates' descendants	Mean^a^		0.00 [− 0.01,0.01]	0.84 [0.79,0.89]	0.61 [0.58,0.64]
		p_value	SBV	0.78	0.03	0.02
			Line	0.22	< 0.01	0.25
			$\mathbf{A}$ or $\mathbf{H}$ matrix	0.76	0.71	0.70
Phenotypes, pedigree information	Phenotypes, pedigree and genomic information	Mean^a^		− 0.08 [− 0.09,− 0.07]	0.96 [0.92,1.00]	0.89 [0.87,0.92]
		p_value	SBV	< 0.01	0.68	0.37
			Line	0.41	0.97	0.61

^a^95% confidence interval in bracket

Bias: ${\Delta \mu }_{wp}=\frac{\overline{{ESBV }_{p}}-\overline{{ESBV }_{w}}}{{\mathbf{L}}_{\widehat{\mathbf{G}},\mathbf{l}}^{\mathbf{^{\prime}}}\widehat{\mathbf{G}}{\mathbf{L}}_{\widehat{\mathbf{G}},\mathbf{l}}}$; dispersion: ${b}_{w,p}=\frac{cov\left({ESBV}_{p},{ESBV}_{w}\right)}{var\left({ESBV}_{p}\right)}$; Ratio of accuracy: ${\rho }_{w,p}=\frac{cov\left({ESBV}_{p},{ESBV}_{w}\right)}{\sqrt{var\left({ESBV}_{p}\right)var\left({ESBV}_{w}\right)}}$

Indices *w* = estimates obtained with more information,$p$ = estimates obtained with less information

**Table 2:** Comparison of SBV predicted using longitudinal phenotypes^a^ and EBV predicted using summarized phenotypes^b^, using pedigree and pedigree plus genomic information for the low (LRFI) and high (HRFI) RFI lines

	Pedigree		Pedigree + genomics	
	LRFI	HRFI	LRFI	HRFI
$Bias$ ${\overline{SBV} }_{w}-\overline{{u }_{ySBVw}}$				
${SBV}_{1}$	0.00	0.02	0.00	0.02
${SBV}_{2}$	0.03	0.00	0.02	0.00
${SBV}_{3}$	0.01	0.00	0.01	0.00
Dispersion ${b}_{{SBV}_{w},{u}_{ySBVw}}$				
${SBV}_{1}$	1.05	1.03	1.04	1.03
${SBV}_{2}$	1.03	1.02	1.03	1.03
${SBV}_{3}$	1.00	1.01	0.97	1.00
Ratio of accuracies ${\rho }_{{SBV}_{w},{u}_{ySBVw}}$				
${SBV}_{1}$	1.00	1.00	1.00	1.00
${SBV}_{2}$	1.00	0.99	0.99	0.99
${SBV}_{3}$	0.98	0.98	0.98	0.98

^a^Longitudinal phenotypes = weekly measurements of FI analyzed with an RR model: ${FI}_{ij}={\mathbf{x}}_{ij}{\varvec{\upbeta}}+{{\beta }_{1}MBW}_{ij}+{\beta }_{2}{ADG}_{ij}+{\beta }_{3}{BFT}_{ij}+\sum_{q=0}^{2}{\mathrm{a}}_{iq}{\varphi }_{q}\left(j\right)+\sum_{q=0}^{2}{\mathrm{b}}_{iq}{\varphi }_{q}\left(j\right)+{e}_{ij}$, ${\mathrm{SBV}}_{\mathrm{l},\mathrm{i}}={\mathbf{L}}_{\mathrm{Gl}}{\mathbf{u}}_{\mathrm{i}}$, where ${\mathbf{L}}_{\mathrm{Gl}}$ is the ${l}^{th}$ eigenvector from the decomposition of the $\mathbf{G}$ matrix

^b^Summarized phenotypes:$${\mathbf{y}}_{{\mathbf{S}\mathbf{B}\mathbf{V}}_{\mathbf{l}}}={\mathbf{L}}_{\widehat{\mathbf{G}},\mathbf{l}}^{\mathbf{^{\prime}}}\left(\mathbf{F}\mathbf{I}-\mathbf{X}\widehat{{\varvec{\upbeta}}}-\widehat{{\beta }_{1}}\mathbf{M}\mathbf{B}\mathbf{W}-\widehat{{\beta }_{2}}\mathbf{A}\mathbf{D}\mathbf{G}-\widehat{{\beta }_{3}}\mathbf{B}\mathbf{F}\mathbf{T}\right)$$

## Discussion

The objectives of the present study were to evaluate, for longitudinal RFI in pigs, the quality of the model predictions for SBV that are useful for selection, and the benefit of adding genomic information. In addition, for practical selection on longitudinal profiles, we evaluated the consequences of using summarized phenotypes instead of longitudinal phenotypes on estimates of (summarized) breeding values. To compare estimates of breeding values obtained in different situations, we applied the LR method proposed by Legarra and Reverter [31]. The use of the LR method in place of traditional cross-validation tests overcomes the limitations of the latter since it does not need “true” BV (i.e. highly accurate EBV) or pre-corrected phenotypes. Thus, the method is particularly suitable for pig populations where selection candidates do not necessarily have own performance or numerous recorded progeny, and it is all the more useful for longitudinal data for which missing data are frequent. In addition, the BV of interest for selection in the longitudinal case, i.e. SBV, are a linear combination of EBV that accumulates those aforementioned difficulties for each time point, making the computation of the accuracy of the resulting SBV with traditional approaches quite complex when information is heterogeneous across time points and candidates. To be relevant, the LR method should be applied to a set of focal individuals that is sufficiently large and diverse (i.e. animals from several families), that should represent the population of interest (selection candidates in our case), for which the quantity of “information” used to estimate SBV should be similar for the different focal individuals in the partial dataset as well as in the whole dataset and for which the reference EBV should be reasonably accurate. In the present study, progeny from all families were candidates to selection, while focal individuals were the genotyped dams used for breeding in the next generation. The dams were randomly selected within sire, one female replacing its dam, to maintain the genetic diversity, so they represent the population of interest and fulfilled the requirement in terms of diversity. Because experimental lines have a limited number of breeding animals, the groups of focal individuals were small and thus did not fulfil the ‘sufficiently large’ requirement. To partially counteract this, we repeated, as Macedo et al. [36], the estimation of LR indicators in successive focal groups. In order to meet the requirement for the focal individuals to have the same quantity of information to predict their EBV, only genotyped dams were considered as focal individuals, and not genotyped sires. Indeed, the quantity of information to predict the SBV of sires and dams would have been similar in the partial dataset (phenotypes from ascendants), but not in the whole dataset, because sires had on average six times more phenotyped progeny than females. Finally, the theoretical accuracy of the reference EBV (whole population) for the focal individuals was around 0.6 for SBV1 and 0.5 for SBV2 and SBV3, and thus fulfilled the requested reasonable accuracy (Andres Legarra, personal communication). In this study, we were not interested in estimating the accuracy of SBV of the focal animals themselves, as the ratio of accuracies was sufficient to judge the quality of the SBV at the time of selection and to evaluate the gain from genomic information for selection. Nevertheless, it is possible to estimate the (selected) reliability of SBV at the time of selection with the LR method by computing $\frac{cov\left({SBV}_{p},{SBV}_{w}\right)}{{\sigma }_{u*}^{2}}$, where ${\sigma }_{u*}^{2}$ is the genetic variance of the group of individuals of interest, which can be estimated by Gibbs sampling [37].

The bias ($${\Delta \mu }_{wp})$$ and dispersion ($${b}_{w,p})$$ obtained when evaluating the quality of predictions of (G)ESBV at the time of selection give indications on the errors that can be made on the expected genetic gain at the stage of selection [31]. On the one hand, the bias was null on average. On the other hand, the (G)ESBV were over-dispersed, especially in the LRFI line, which is in line with the difference in dispersion between the two lines reported for the same population by Aliakbari et al. [38]. This over-dispersion results in overestimation of the expected genetic gain (by $$\simeq 0.2{\sigma }_{SBV}$$, [31]). As expected, ESBV of the focal individuals predicted without phenotypes from descendants were significantly less accurate than ESBV predicted using all the phenotypic information ($${\rho }_{w,p}$$ significantly lower than 1). We did not detect differences in bias, dispersion, and relative gain in accuracy due to additional phenotypes, between ESBV obtained using genomic and pedigree information or pedigree information only. Yet, it is expected that “*additional phenotypic records would have lower impact on GEBV (*compared to EBV*) because they would contribute with less information than the direct genomic value*” [39]. The lack of effect of genomic information on SBV prediction in the present study is confirmed by the high correlation between ESBV and GSBV and the high regression coefficient of GSBV on ESBV that we obtained. In our study, the small benefit from genomic information is likely due to the small number of available genotyped animals with accurate phenotype information, much smaller than the expected number (1690, [40]) that is necessary to represent the genomic structure of the population [41]. Consequently, too little of the genetic variance could be captured by the genomic information in this dataset to have a significant impact on ESBV. In addition, there were no animals with both genotype and phenotype, and the number of phenotyped progeny for each focal genotyped animal was small. It should be noted that a single set of scaling factors to construct the $$\mathbf{H}$$ matrix has been tested, although their values may impact bias and dispersion of GEBV [42].

Breeding values that summarized the trajectory of RFI over time using eigenvectors of the estimated variance–covariance structure of the longitudinal data were obtained using two approaches: ${SBV}_{l}$, which were extracted from the genetic analysis of the longitudinal phenotypes (considered as the reference), and ${u}_{{SBV}_{l}}$, which corresponds to the breeding values in the analysis of summarized phenotypes. The latter requires much less computing time, and thus is more suitable for routine evaluation. When, in the model for summarized phenotypes ${y}_{SBV}$, the longitudinal phenotypes are pre-corrected for fixed effects and the variance components are assumed known and fixed, our results (Table 2) show that these two approaches give exactly the same EBV. In practice, pre-correction for all fixed effects is not always possible (i.e. contemporary group effects). To evaluate the additional noise generated in such a situation, we performed the same comparison between ${SBV}_{l}$ and ${u}_{{SBV}_{l}}$ using summarized phenotypes that were not corrected for fixed effects, except for regression on other phenotypes to obtain RFI (i.e. ${\mathbf{y}}_{{{\varvec{S}}{\varvec{B}}{\varvec{V}}}_{{\varvec{l}}}}={\mathbf{L}}_{\widehat{\mathbf{G}},{\varvec{l}}}^{\mathbf{^{\prime}}}\left(\mathbf{F}\mathbf{I}-\widehat{{\beta }_{1}}\mathbf{M}\mathbf{B}\mathbf{W}-\widehat{{\beta }_{2}}\mathbf{A}\mathbf{D}\mathbf{G}-\widehat{{\beta }_{3}}\mathbf{B}\mathbf{F}\mathbf{T}\right)$), but by including and estimating the effect of the fixed effects in the model used for their analysis (the same fixed effects as used in the longitudinal model except for time and interactions with time). This analysis resulted in similar bias and dispersion as those obtained when pre-correcting phenotypes for all fixed effects. The correlations between ${SBV}_{l}$ and ${u}_{{ySBV}_{l}}$ were slightly lower but still large for ${\mathrm{SBV}}_{1}$ (0.92 and 0.95 for LRFI and HRFI, respectively) and ${\mathrm{SBV}}_{2}$ (0.94 for both lines), but lower for ${\mathrm{SBV}}_{3}$ (0.78 for both lines). Since the first two SBV have been identified as sufficient to select for a desired trajectory pattern of RFI over time in pigs [32], using the model on summarized phenotypes seems a good alternative (similar accuracy with less computing time) to select for RFI trajectories in routine evaluation.

## Conclusions

Using the LR method, we evaluated, the quality of prediction of breeding values for candidates without own phenotypes in the study of longitudinal data, by using two different approaches (analysis of longitudinal or summarized phenotypes), and evaluated the benefit of adding genomic information for prediction. Predictions were of similar quality with the two approaches, meaning that, in this population design, the use of summarized phenotypes would be of interest for routine evaluation of longitudinal traits. We did not highlight any benefit of genomic information for prediction in this study, which is certainly due to the number of genotyped animals with accurate estimation being too small in this dataset, contrary to the usual expectation.

## Supplementary Information

**Additional file 1: Table S1.** Descriptive statistics of the data. **Table S2.** Numbers of records, animals with phenotype, sires, and dams per line and generation

**Additional file 2: Figure S1.** Ls means of the line effect on the LR statistics ${b}_{w,p}$ (left panel) and of the type of SBV on ${b}_{w,p}$ (middle panel), and ${\rho }_{w,p}$ (right panel) evaluating the quality of the model to predict SBV based on phenotypic information on ascendants and collateral relatives of the focal individuals. **Figure S2.** Ls means of the type of SBV on the LR statistics $\Delta {\mu }_{w,p}$, evaluating the quality of the model to predict SBV based on phenotypic and pedigree information.

## Data Availability

The datasets analysed during the current study are available from Hélène Gilbert (Helene.gilbert@inrae.fr) on reasonable request.
