# Supplementary material for: Quality of breeding value predictions from longitudinal analyses, with application to residual feed intake in pigs
Source: Genet Sel Evol. 2022 May 13;54:32. doi: 10.1186/s12711-022-00722-w (PMC9103455; doi:10.1186/s12711-022-00722-w)
Supplement: Supplementary file 1 — Additional file 1: Table S1. Descriptive statistics of the data. Table S2. Numbers of records, animals with phenotype, sires, and dams per line and generation [file 12711_2022_722_MOESM1_ESM.pdf]

Additional file 1 : Descriptive statistics and number of records

Supplementary Table1 : Descriptive statistics of the data

**Week**	**ADG (g/d)**	**MBW ($Kg^{0.6}$)**	**BFT (mm)**	**FI (Kg/d)**
	**Mean±sd**	**Mean±sd**	**Mean±sd**	**Mean±sd**
1	746±132	9.2±0.8	8.8±1.5	1.68±0.34
2	776±120	9.9±0.8	9.3±1.7	1.80±0.35
3	787±113	10.6±0.8	9.9±1.8	1.93±0.35
4	815±122	11.3±0.9	10.4±1.9	2.02±0.37
5	844±135	11.9±0.9	10.9±2.0	2.12±0.37
6	841±126	12.6±0.9	11.4±2.2	2.20±0.38
7	851 ±134	13.2±0.9	11.9±2.4	2.29±0.39
8	856±147	13.8±0.9	12.5±2.5	2.36±0.40
9	832±161	14.4±0.9	13.1±2.6	2.43±0.40
10	825±169	15.0±0.9	13.5±2.8	2.45±0.40

ADG = average daily gain, MBW = metabolic body weight, BFT = backfat thickness, FI = feed intake

Supplementary Table 2: Numbers of records, animals with phenotype, sires, and dams per line and generation

generation	line	number of observations	number of animals with phenotype	number of sires	number of genotyped sires*	number of dams	number of genotyped dams*
G0		753	80	28		30	
G1	LRFI	718	74	6	6	35	35
G1	HRFI	815	84	6	6	40	39
G2	LRFI	1,355	143	6	6	34	33
G2	HRFI	1,377	145	6	6	42	38
G3	LRFI	1,579	163	6	6	40	39
G3	HRFI	1,313	138	6	6	41	41
G4	LRFI	1,651	173	6	5	41	41
G4	HRFI	1,456	155	6	5	43	42
G5	LRFI	1,748	179	6	6	44	41
G5	HRFI	1,470	150	6	5	39	37
G6	LRFI	2,057	211	6	5	40	40
G6	HRFI	1,691	174	6	6	48	47
G7	LRFI	2,636	274	6	6	38	37
G7	HRFI	2,334	254	6	6	35	34

*Animals with validated genotypes

Sires and dams correspond to the parents of the animals with phenotype, i.e. in G1, the 74 animals with phenotype are offspring of 6 sires and 35 dams.
