# Supplementary material for: Quality of breeding value predictions from longitudinal analyses, with application to residual feed intake in pigs
Source: Genet Sel Evol. 2022 May 13;54:32. doi: 10.1186/s12711-022-00722-w (PMC9103455; doi:10.1186/s12711-022-00722-w)
Supplement: Supplementary file 2 — Additional file 2: Figure S1. Ls means of the line effect on the LR statistics ${b}_{w,p}$bw,p (left panel) and of the type of SBV on ${b}_{w,p}$bw,p (middle panel), and ${\rho }_{w,p}$ρw,p (right panel) evaluating the quality of the model to predict SBV based on phenotypic information on ascendants and collateral relatives of the focal individuals. Figure S2. Ls means of the type of SBV on the LR statistics $\Delta {\mu }_{w,p}$Δμw,p, evaluating the quality of the model to predict SBV based on phenotypic and pedigree information. [file 12711_2022_722_MOESM2_ESM.pdf]

Additional file 2: LSMeans of the LR statistics evaluating quality of the prediction of SBV

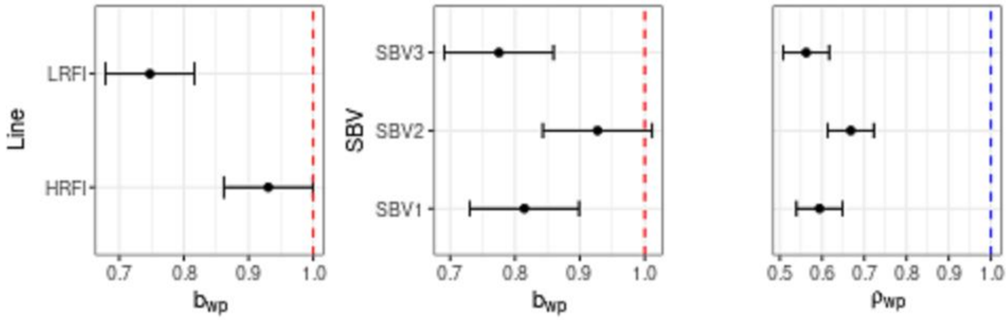

Figure S1: Ls means of the line effect on the LR statistics $b_{w,p}$ (left panel) and of the type of SBV on $b_{w,p}$ (middle panel), and $\rho_{w,p}$ (right panel) evaluating quality of the model to predict SBV based on phenotypic information on ascendants and collateral relatives of the focal individuals.

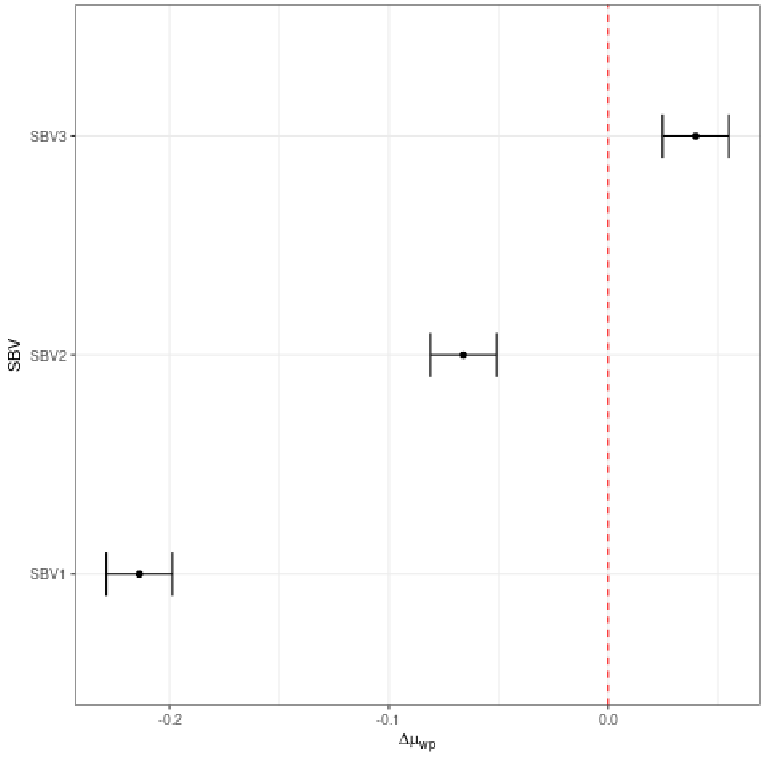

Figure S2: Ls means of the type of SBV on the LR statistics $\Delta\mu_{wp}$, evaluating quality of the model to predict SBV based on phenotypic and pedigree information
